# Anxiety-Depressive Disorders in 200 Patients with Post-COVID-19 Syndrome: Prevalence and Predictors from a Cross-Sectional Study

**DOI:** 10.3390/medicina62071234

**Published:** 2026-06-26

**Authors:** Sylwia Drzymała, Anna Blask-Osipa, Anna Szczepańska-Alvarez, Hanna Markowska, Małgorzata Dobrzyńska, Sławomira Drzymała-Czyż, Jarosław Walkowiak

**Affiliations:** 1 Department of Pediatric Gastroenterology and Metabolic Diseases, Poznan University of Medical Sciences, Szpitalna 27/33, 60-572 Poznan, Poland; jarwalk@ump.edu.pl; 2 Complex of Healthcare Institutions, Kościuszki 96, 64-700 Czarnków, Poland; annablask@wp.pl (A.B.-O.); hmarkowska8@gmail.com (H.M.); 3 Department of Mathematical and Statistical Methods, Poznan University of Life Sciences, 60-637 Poznan, Poland; anna.szczepanska-alvarez@up.poznan.pl; 4 Department of Bromatology, Poznan University of Medical Sciences, Marcelińska 42, 60-354 Poznan, Poland; mdobrzynska@ump.edu.pl (M.D.); drzymala@ump.edu.pl (S.D.-C.)

**Keywords:** post-COVID-19 syndrome, hospital anxiety and depression scale, beck depression inventory

## Abstract

*Background and Objectives*: Recovering from COVID-19 does not always imply a full return to health and may lead to the development of post-COVID-19 syndrome. Post-COVID-19 manifestations include, among others, symptoms of depression and/or anxiety. The aim of the study was to assess the prevalence of anxiety and depressive disorders and to identify their exogenous and endogenous predictors in individuals with post-COVID-19 syndrome. *Materials and Methods*: The study included 200 participants (116 women and 84 men, aged 18–80) diagnosed with post-COVID-19 syndrome. Participants completed psychological assessments, including the Hospital Anxiety and Depression Scale (HADS), the Generalized Anxiety Disorder 7 (GAD-7), and the Beck Depression Inventory (BDI). Comorbidities were also evaluated. *Results*: Based on the HADS, anxiety was identified in 41.5% of respondents and depression in 39.5%. Generalized anxiety disorder was screened positive for 36.5% of respondents (GAD-7), while mild depression was observed in 37.0% (BDI). Among participants with post-COVID-19 syndrome and diabetes, the risk of developing depression was three times higher than in individuals without comorbidities. In smoking women with post-COVID-19 syndrome and diabetes, the risk of developing depressive disorders was estimated to exceed 90%. *Conclusions*: The risk of developing anxiety and depressive disorders in individuals with post-COVID-19 syndrome and multimorbidity is very high, highlighting the need for preventive psychological care, including targeted screening programs, for those at greatest risk.

## 1. Introduction

From March 2020 to 8 January 2025, over 6,770,134 confirmed SARS-CoV-2 infections have been recorded in Poland, suggesting that the true number of cases may reach tens of millions [1]. Often, contracting COVID-19 does not result in complete recovery, and many patients reported persistent symptoms. Symptoms that develop during or after COVID-19, persist for more than 12 weeks, and cannot be explained by another diagnosis are classified as post-COVID-19 syndrome [2]. The most common post-COVID-19 manifestations include fatigue (58%), headaches (44%), impaired concentration (27%), hair loss (25%), shortness of breath (24%), taste disturbances (23%), and loss of smell (21%) [3,4]. Fifty-five potential long-term symptoms associated with COVID-19 have been defined [5], among which neuropsychiatric symptoms deserve particular attention. Chronic neuropsychiatric consequences of SARS-CoV-2 infection include depression, anxiety, psychotic disorders, and demyelinating or neuromuscular complications [6]. These manifestations are associated with greater functional impairment and an overall decline in quality of life [7]. A particular group of patients at risk of these complications are those with multiple comorbidities and chronic inflammation who receive medications that may have antagonistic effects and in whom anxiety and depressive disorders are increasingly reported [8,9,10].

Several hypothetical mechanisms underlying neuropsychiatric manifestations in post-COVID-19 syndrome have been proposed, including immune dysregulation, cytokine imbalance, thrombosis, and disturbances of the gut–brain axis [11,12]. Persistent gastrointestinal SARS-CoV-2 shedding and post-infectious dysbiosis may also contribute to chronic inflammation and altered neurotransmitter signaling [13,14]. However, these mechanisms remain incompletely understood and were not directly investigated in the present study.

Current studies mainly describe the frequency of stress experienced during the COVID-19 pandemic, attributing it to factors such as stricter epidemic restrictions (e.g., limited direct contact, remote learning, isolation, quarantine, etc.) [15,16,17,18] or the fear of illness and death [19,20]. However, most research focuses on pandemic-related distress rather than on clinically relevant anxiety depressive disorders in individuals with post-COVID-19 syndrome. Evidence on factors that increase vulnerability to these disorders in the post-COVID-19 population remains scarce, despite the heterogeneity and clinical complexity of this group. Other studies address long COVID, a broad term encompassing all symptoms persisting after acute SARS-CoV-2 infection [21,22].

While mental disorders are increasingly recognized as a complication of COVID-19, there remains a lack of data on exogenous and endogenous predictors, including specific demographic and clinical factors that may predispose individuals to anxiety-depressive disorders in post-COVID-19 syndrome. A better understanding of such risk factors is essential for improving early detection and targeted clinical management. Therefore, the aim of the present study was to assess the prevalence of anxiety and depressive disorders and to identify their exogenous and endogenous predictors in individuals with post-COVID-19 syndrome. This study was conducted to provide preliminary data and to explore potential predictors of anxiety depressive disorders rather than to establish their prevalence.

## 2. Materials and Methods

**Figure 1:**
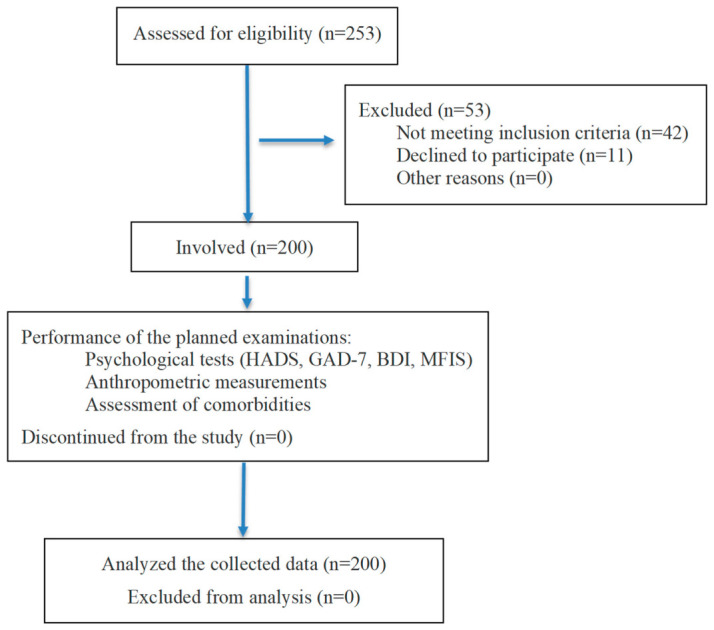
Participants flow diagram.

The flowchart of the study is depicted in Figure 1. More than 250 individuals expressed interest in participating in the study. Ultimately, the study involved 200 participants (116 women and 84 men, aged 18 to 80) admitted to the Health Care Complex post-COVID ward in Czarnków, an infectious disease hospital, until April 2022. Participants were recruited, according to the inclusion and exclusion criteria, from patients admitted to a specialized post-COVID ward at an infectious disease hospital. The department admitted participants who fulfilled the National Institute for Health and Care Excellence (NICE) criteria for post-COVID-19 syndrome, i.e., persistence of symptoms for more than 12 weeks after acute SARS-CoV-2 infection [23]. In practice, participants were admitted for post-COVID-19 evaluation as soon as possible after referral. Approximately 90% of participants were assessed during the fifth month after acute infection, whereas the remaining participants were evaluated during the sixth month. Additional inclusion criteria for the study were age ≥ 18 years and consent to participate. The exclusion criteria included pregnancy and an actively treated form of a previously diagnosed (pre-COVID-19) psychiatric disorder and current use of psychotropic medications, the use of stimulatory supplements/medications, and drug addiction. Due to the screening and epidemiological design, the sample covered a wide age range.

This paper presents research carried out between 2021 and 2022.

Post-COVID-19 syndrome symptoms were assessed in each participant according to the guidelines of the National Institute for Health and Care Excellence (NICE) [2]. Anthropometric parameters were measured in all study participants, and body mass index (BMI) was calculated. Based on previous test results provided or performed during hospitalization, study participants were characterized for the presence of hypertension, ischemic heart disease, diabetes, chronic obstructive pulmonary disease, inflammatory bowel disease, any chronic thyroid disease, chronic liver disease, pre-existing mental illness diagnosed before the COVID-19 pandemic, and addictions (tobacco smoking, alcohol dependence, and gambling).

### 2.1. Psychological and Fatigue Assessments Included

- Hospital Anxiety and Depression Scale (HADS) assesses the intensity of anxiety and depressive symptoms [24]. It is a 16-item questionnaire using a four-point Likert scale (0–3) assessing anxiety (8 items) and depression (8 items) during the last week. Scores of 0–7 indicate normal results, 8–10 represent borderline levels, and 11–21 indicate abnormal results (confirming depression and/or anxiety).
- Generalized Anxiety Disorder-7 Questionnaire (GAD-7) assessed the degree of anxiety [25]. It is a 7-item questionnaire assessing anxiety symptoms over the past 14 days. Scores > 10 were considered indicative of clinically relevant generalized anxiety symptoms and a positive screening result for possible generalized anxiety disorder.
- Beck Depression Inventory (BDI), measuring mental well-being [26,27], is a 21-item questionnaire assessing depressive symptoms over the past two weeks. Cut-off points: mild (12–26), moderate (27–49), severe (50–63).
- Modified Fatigue Impact Scale (MFIS) assesses the degree of fatigue impact [28,29], a 21-item questionnaire assessing fatigue impact on physical, cognitive, and psychosocial functioning. Total score range: 0–84; significant impact indicated by scores > 38.

All instruments used in the study (HADS, GAD-7, and BDI-II) have been validated in Polish populations and demonstrate good to very good psychometric properties. The Polish version of the HADS shows satisfactory internal consistency, with Cronbach’s α values ranging from approximately 0.78 to 0.84 for its subscales, although slightly lower reliability has been reported for the depression component [30]. The Polish adaptation of the GAD-7 demonstrates high internal consistency and unidimensional structure, with Cronbach’s α typically around 0.90, confirming its reliability as a screening tool for generalized anxiety [31]. Similarly, the Polish version of the Beck Depression Inventory-II (BDI-II) demonstrates very good psychometric properties, including high internal consistency and validity comparable to those of the original instrument, supporting its use in both clinical and research settings [32].

Psychological assessment tests were chosen to capture complementary aspects of psychological functioning. The HADS was used as a screening tool for general anxiety and depression symptoms in the medical population, minimizing the confounding influence of somatic symptoms. The GAD-7 provided a more detailed evaluation of generalized anxiety severity, while the BDI allowed for a more detailed assessment of depressive symptomatology. The use of multiple psychometric tools assessing overlapping constructs (HADS, GAD-7, and BDI) may have introduced redundancy in the assessment of anxiety and depressive symptoms. However, this approach was intended to enhance the robustness of the findings through cross-validation across instruments with slightly different sensitivities and clinical focuses. This approach is consistent with other studies [33,34,35].

The research followed the authors’ instructions for the psychological tests. All tests were conducted on hospital premises in a separate room, with only the patient and the psychologist (SD) present. The psychologist explained the study’s purpose and assured participants that their responses would remain confidential. If participants made comments or asked questions, the psychologist explained their doubts. No time limits have been introduced. After completing the questionnaire, the examiner checked whether the participants answered all the questions.

### 2.2. Sample Size Calculation

The calculation of the minimum sample size was based on the prevalence of anxiety and depressive disorders. The sample size estimation assumed that the population proportion of psychosocial symptoms in long COVID was 47.8% [36], and that the difference between the population proportion and the examined proportion would be 10%. These assumptions were supported by preliminary results from a study describing the prevalence of anxiety disorders in participants with long COVID. The minimum sample size was determined using the GPower 3 software (University of Düsseldorf, Germany). Assuming a power of 80%, a two-sided α of 0.05, and an effect size (g) of 0.1, the calculated minimum sample size was 198 participants.

### 2.3. Statistical Analysis

The results of the anthropometric and psychological parameters were expressed as medians and the 1st–3rd quartile ranges. Differences between participants with and without disease were assessed using the Mann–Whitney test. A value of *p* < 0.05 was considered statistically significant.

Based on psychological tests, participants were divided into two groups: those who exhibited symptoms of depression during post-COVID-19 syndrome and those who did not. The criterion confirming the occurrence of anxiety-depressive disorders was a score above 10 points for the following tests: Hospital Anxiety and Depression Scale, Generalized Anxiety Disorder-7, and above 11 points for the Beck Depression Inventory test.

The presence of a coexisting disease was determined on a binary scale, meaning: 0 = no disease, 1 = disease present. Based on Fisher’s exact test, the relationship between the obtained results of various psychological tests was examined, and the relationship between the results of appropriate psychological tests and comorbidities, participants’ gender, and addictions was verified. Moreover, a logit model was used to examine the impact of comorbidities on the risk of developing depression after SARS-CoV-2 infection [37,38].

$$\ln\frac{p_{i}}{1-p_{i}}\;=\;\beta_{0}+\sum_{j=1}^{k}\beta_{j}x_{ij}$$

where $\frac{p_{i}}{1-p_{i}}$ is the odds ratio, the quotient of the probability of developing depression by the probability of not suffering from depression in the i-th patient, x_ij_ is a binary variable indicating the existence of the j-th disease in the i-th patient, j = 1, 2, …, k, while β_0_, β_j_ are unknown parameters. Variable selection was performed using a two-step approach. First, univariate analyses (Fisher’s exact test and unadjusted logistic regression, as appropriate) were conducted to assess the association between each potential predictor and the outcome variable. Variables demonstrating a potential association (*p* < 0.10) were subsequently considered for inclusion in the multivariable model. Prior to model construction, multicollinearity among predictors was assessed, and highly correlated variables were excluded to avoid redundancy and model instability. The final multivariable logistic and linear regression models included variables identified during the univariate screening procedure. Model fit was assessed by comparison with the null (intercept-only) model using the Likelihood Ratio Test, and model performance was evaluated using the Akaike Information Criterion (AIC). Detailed results of the univariate analyses, variable selection procedure, model parameters, and goodness-of-fit indices are provided in the Supplementary Materials. All statistical analyses were performed at a significance level of 0.05.

Statistical analysis was performed using GraphPad Prism 5.01 software (GraphPad Software, Inc., La Jolla, CA, USA), Statistica 13.0 software (TIBCO, Palo Alto, CA, USA), and the R 4.3.1 program.

### 2.4. Ethical Considerations

The consent to conduct the study was obtained from the Bioethics Committee of Poznan University of Medical Sciences (665/21). The study was conducted in accordance with the Declaration of Helsinki [39].

## 3. Results

The anthropometric and clinical characteristics of the study participants are presented in Table 1 and Table 2. The median age of the participants was 57 years (range 18–80 years), 58% were women. The largest proportion of respondents was aged 50–59 years (32.5%) and 60–69 years (28.5%). The median BMI value was 29.5 kg/m^2^, and the largest group of hospitalized participants was overweight and obese (33.5% and 45%, respectively). The mean time between acute COVID-19 and study assessment was 5.1 ± 0.3 months; median (interquartile range): 5 (5–5).

The most common comorbidities were hypertension, thyroid disease, and ischemic heart disease. Interestingly, only 7.5% of hospitalized participants admitted to smoking tobacco regularly. Among other addictions, only alcohol dependence was reported.

The results of psychological assessments evaluating clinical symptom severity, medication impact, and overall well-being are presented in Table 3. The median HADS scores were 8 for anxiety and 9 for depression. According to this test, anxiety was confirmed in 41.5% of respondents, while depression was observed in 39.5%.

The median score of the GAD-7 test was 7. Screening positive for generalized anxiety disorder (GAD-7 score > 10) was observed in 36.5% of respondents, of which 2.0% of participants obtained the highest possible level (21 points out of 21 possible).

The median BDI score was 11. Mild depression was identified in 37.0% of respondents (score ranging from 12–26 points), and moderate depression in 9.0% (over 27 points).

The median total score in the MFIS test was 35 points. A score of 38 or higher was recorded in 45% of respondents.

The results of psychological tests in participants with post-COVID-19 syndrome, depending on various clinical parameters, are presented in Table 4. Diseases and alcohol dependence present in more than 5% of participants were included in the analysis. Anxiety, as assessed by the HADS and GAD-7 tests, and depression, as measured by the BDI test, were more frequently observed in women. Depressive symptoms were more commonly reported in individuals with ischemic heart disease. However, severe fatigue occurred more often in participants who were overweight or obese and in participants without thyroid diseases. Anxiety-depressive disorders, identified through multiple psychological assessments, were more prevalent among individuals with diabetes and nicotine addiction.

Analysis of binary features using Fisher’s exact test confirmed relationships between psychological test results in Table 5 (for more details, see Supplementary Tables S1 and S2). Additionally, smoking, diabetes, pre-COVID-19 psychiatric disorders, and gender were significantly associated with the risk of depression in post-COVID-19 syndrome. This relationship was different for the psychological tests used in the study.

**Figure 2:**
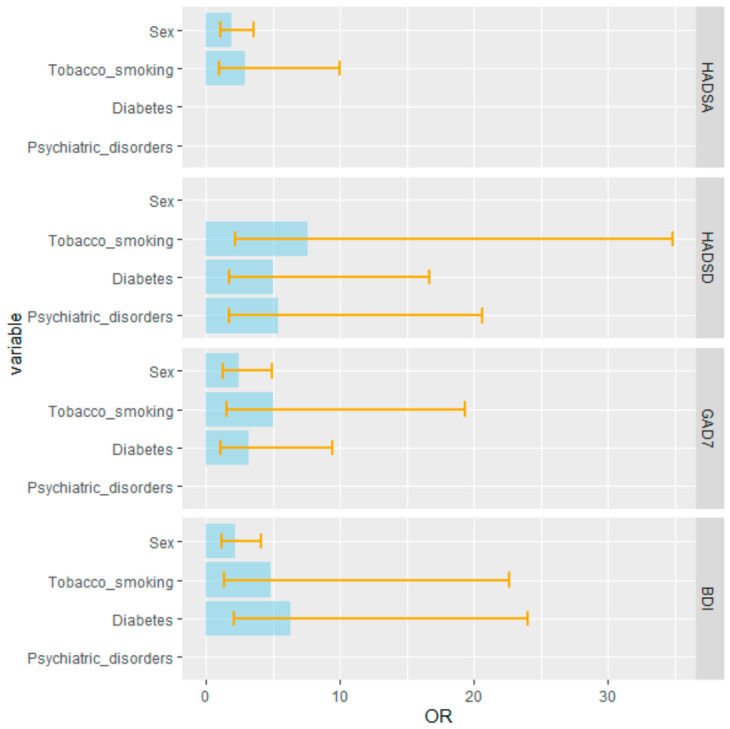
The odds ratio with 95% confidence intervals. HADS—Hospital Anxiety and Depression Scale; GAD-7—Generalized Anxiety Disorder-7; BDI—Beck Depression Inventory.

The relationship between all features included in the logit model was statistically significant, and based on the results obtained, the risk of developing depression during the post-COVID-19 syndrome was determined. Figure 2 shows the logarithmic scale of the analyzed data: OR and confidence interval (for details, see Supplementray Tables S4a, S5a and S6a).

In smokers with post-COVID-19 syndrome, the odds of anxiety disorders, as assessed by the HADS-anxiety psychological test, were found to be almost three times higher. Logistic model analysis demonstrated that smoking, diabetes, and pre-existing psychiatric disorders significantly increase the odds of developing depression in the course of post-COVID-19 syndrome, as measured by the HADS-depression test. Specifically, participants with post-COVID-19 syndrome and diabetes had a fivefold higher odd of depression; those with pre-existing psychiatric disorders had an approximately 5.5-fold higher odds; and smokers had nearly a 7.5-fold higher odds.

Using the GAD-7 test, individuals with post-COVID-19 syndrome and diabetes were documented to have a threefold increased odds of anxiety compared with those without comorbidities; in smokers, the odds were fivefold higher, and women with post-COVID-19 syndrome had a 2.5-fold increased odds. The odds ratio for depression developing, assessed by BDI, was approximately 6 in participants with post-COVID-19 syndrome and diabetes, and approximately 5 in smokers. Across the GAD-7, HADS-anxiety and BDI results, women with post-COVID-19 syndrome had approximately twice the odds ratio of anxiety compared with men.

Using the adopted logistic model that took into account the impact of comorbidities, the probability of developing anxiety-depressive disorders after SARS-CoV-2 virus infection was estimated (Table 6). As with previous models, the coexistence of diabetes and tobacco dependence was confirmed to be a significant predictor of anxiety and depressive disorders in participants with post-COVID-19 syndrome. The probability of anxiety, as assessed by the GAD-7, was approximately 81% in men and 91% in women; defined by the BDI, it was approximately 93% in men and 96% in women, respectively.

## 4. Discussion

The main findings of this study indicate that individuals with post-COVID-19 syndrome who also have non-communicable diseases and/or nicotine addiction may be at higher risk of anxiety-depressive disorders.

It is worth emphasizing that our study did not aim to determine the prevalence of anxiety–depressive disorders in the population that had COVID-19, but to estimate the odds of these disorders occurring in individuals who developed post-COVID-19 syndrome. Previous studies have described the onset of anxiety and depression in post-COVID-19 patients with potential correlations to factors such as systemic inflammation (elevated CRP levels, altered neutrophil-to-lymphocyte ratio, and decreased monocyte-to-lymphocyte ratio) [40,41]. Other research has associated lower education and previously occurring migraines (primarily worse cognitive outcomes) [42,43]. Additionally, exposure to earlier traumatic events has been highlighted as a relevant factor, as post-COVID-19 disorders may resemble, though not fully meet criteria for, post-traumatic stress disorder [44,45]. However, the extent to which comorbidities influence the development of these disorders, as well as the precise risk associated with anxiety-depressive conditions in post-COVID-19 syndrome, remains undefined. Addressing this gap is a key novelty of our manuscript.

The results of our study indicate that the co-occurrence of comorbidities, particularly type 2 diabetes, was associated with a higher likelihood of anxiety and depressive disorders. Our findings align with previous studies that also reported a higher incidence of depression in individuals with diabetes, highlighting the existence of a vicious cycle of mental health disorders in this patient group [46,47,48]. This cycle occurs because individuals with depression are more likely to develop diabetes, while those with diabetes experience a faster onset of depression. Furthermore, diabetes increases the likelihood of developing a severe form of SARS-CoV-2 infection, which in turn may be associated with anxiety and depressive disorders [46]. However, it is important to note that the study by Fernández-de-Las-Peñas et al. [49] did not find that diabetes predisposes individuals to increased post-COVID-19 mental health symptoms. Unlike our study, which examined participants who had experienced post-COVID-19 complications, their research focused solely on diabetics and the general population who had suffered from COVID-19, assessing whether complications arose at all.

Our findings showed that women had higher odds ratios of anxiety and depressive disorders. Global epidemiological studies also demonstrate greater susceptibility of women to depressive disorders; sex differences are more frequently observed among adolescents, whereas among older cohorts, they tend to be more pronounced in societies characterized by greater gender equality [50]. An analysis of available scientific evidence on post-COVID-19 mental disorders in women shows that most research focuses on pregnant or postpartum individuals [51,52,53]. However, some findings suggest that the cyclical nature of women’s hormonal balance may strongly stimulate IgG antibody synthesis, potentially prolonging disease symptoms [54,55]. Conversely, studies emphasize that men over the age of 50 who have had COVID-19 tend to experience lower levels of depressive symptoms compared to women [56].

This study also revealed that smoking may be associated with a higher likelihood of depressive symptoms (BDI) and anxiety (HADS—anxiety and GAD-7). It is worth noting that the percentage of participants who identified as active smokers in our study (7.5%) may be somewhat uncertain, as the participants themselves defined their smoking status as chronic smokers. However, it is tobacco addiction—rather than occasional smoking, as we assume—that may play a significant role in the onset of mood disorders. This perspective is also supported by Sher [57], who, in his study on the impact of smoking on suicide risk among individuals recovering from SARS-CoV-2 infection, emphasizes that the COVID-19 pandemic has led to an increase in tobacco consumption, as smoking is often used as a coping mechanism for stress, anxiety, and depression. At the same time, smoking may exacerbate depression, suicidal thoughts, or even suicide attempts. A meta-analysis of studies conducted between 1966 and 2011 further demonstrated that smoking significantly increases the risk of suicide, with the risk rising for individuals who smoke at least ten cigarettes per day [58].

The increase in the likelihood of anxiety and depressive disorders among participants with post-COVID-19 syndrome was also associated with the presence of previously diagnosed (pre-COVID-19) psychiatric disorders. A meta-analysis published in 2024, which examined the incidence of depression, anxiety, and sleep disorders in individuals recovering from SARS-CoV-2 infection, identified several risk factors for mental health conditions [59]. Demographic factors contributing to their occurrence included older age, female gender, low socio-economic status, and pre-existing psychiatric disorders. Clinical factors increasing the likelihood of mental disorders encompassed the severity of the initial COVID-19 infection and the presence of comorbidities. However, it appears that it is not the severity of the infection itself but rather its long-term consequences, manifested as post-COVID-19 syndrome, that predispose individuals to anxiety and depressive disorders.

Estimated probability analyses suggested that smoking women with post-COVID-19 syndrome and diabetes may have the highest likelihood of developing anxiety and depression. Nevertheless, this finding requires cautious interpretation given the small size of this subgroup.

The present study has several limitations that should be acknowledged. First, the study had a cross-sectional design and did not include longitudinal follow-up; therefore, causal relationships and the long-term trajectory of anxiety-depressive symptoms could not be established. This is a cross-sectional study, the findings reflect statistical associations rather than predictive relationships. Second, baseline psychiatric assessment prior to SARS-CoV-2 infection was unavailable, which limits the ability to determine the extent to which the observed psychological symptoms were directly attributable to post-COVID-19 syndrome. Although participants with actively treated psychiatric disorders and current psychotropic medication use were excluded, subclinical or previously undiagnosed mental health conditions may still have influenced the results. Another limitation is the single-center recruitment from a specialized post-COVID-19 ward of an infectious disease hospital. As mentioned earlier, patients were recruited from a hospital-based post-COVID ward rather than from the general population. Therefore, the study population may not be fully representative of all COVID-19 survivors, as individuals requiring further hospital-based follow-up may have experienced a more severe acute course of illness and/or more persistent post-COVID-19 symptoms. In addition, psychological outcomes were assessed using self-reported psychometric questionnaires rather than structured psychiatric interviews, which may increase the risk of reporting bias. Recall bias should also be considered, particularly with regard to self-reported symptoms and medical history. Furthermore, the study did not include biological or inflammatory markers that could potentially help explain mechanistic links between post-COVID-19 syndrome and anxiety-depressive disorders. Vaccination status and the number of vaccine doses were also not analyzed, although these factors may influence both the severity of acute infection and the risk of long-term complications. Finally, some subgroup analyses involved relatively small sample sizes, particularly for smokers, participants with diabetes, and participants with pre-existing psychiatric disorders, which may have affected the precision of risk estimates and widened confidence intervals.

Nevertheless, despite these limitations, the study provides clinically relevant preliminary data regarding psychological complications in participants with post-COVID-19 syndrome and identifies potentially important high-risk subgroups requiring targeted psychological screening and preventive care.

## 5. Conclusions

The COVID-19 pandemic has affected the mental health of societies worldwide due to the associated mental and physical stress. Many participants who contracted the SARS-CoV-2 virus developed post-COVID-19 syndrome, which was associated with long-term anxiety and depressive symptoms. Smoking, female sex, and comorbidities, particularly diabetes, may be associated with a higher likelihood of anxiety and depressive disorders during post-COVID-19 syndrome, suggesting that individuals at higher risk may benefit from preventive psychological care and targeted screening programs.

## Figures and Tables

**Table 1:** Anthropometric parameters of participants with post-COVID-19 syndrome.

Clinical Parameters	Median	1st–3rd Quartile
Sex ratio [F/M]	116/84	
	(58%)	
Age	57.0	46.0–65.0
female	56.0	45.7–65.0
male	57.0	46.0–64.0
Body height [m]	1.68	1.62–1.73
female	1.65	1.62–1.69
male	1.74	1.72–1.79
Body weight [kg]	79.5	68.3–93.8
female	75.0	63.9–84.6
male	96.2	82.7–109.8
BMI [kg/m^2^]	29.5	25.7–32.5
female	28.8	23.6–30.8
male	30.9	28.3–34.8

BMI—body mass index.

**Table 2:** Clinical data of participants with post-COVID-19 syndrome.

Clinical Parameters	Prevalence (%)	[Yes/No]
Hypertension	48.5	97/113
Ischemic heart disease	12.0	24/176
Diabetes	9.5	19/181
Chronic obstructive pulmonary disease	2.5	5/195
Inflammatory bowel disease	5.0	10/190
Any chronic thyroid disease	17.5	35/165
Any chronic liver disease	3.5	7/193
Pre-existing psychiatric disorders	5.0	10/190
Alcohol dependence	4.0	8/192
Tobacco smoking	7.5	15/185

**Table 3:** Results of psychological and fatigue tests of participants with post-COVID-19 syndrome.

Test	Median	1st–3rd Quartile	% of Results Above the Reference Range
HADS—anxiety	8	2–12	41.5
HADS—depression	9	4–12	39.5
GAD-7	7	2–11	36.5
BDI	11	5–20	46.0
MFIS	35	20–48	45.0

HADS—Hospital Anxiety and Depression Scale; GAD-7—Generalized Anxiety Disorder-7l; BDI—Beck Depression Inventory; MFIS—Modified Fatigue Impact Scale.

**Table 4:** Results of psychological tests of post-COVID-19 syndrome participants defined by different clinical parameters.

Parameters		N	HADS—Anxiety		HADS—Depression		GAD-7		BDI		MFIS	
			Median (1st–3rd Quartiles)									
Age	≤40	27	11	(2.5–12.0)	8	(3.5–13.0)	9	(3.0–12.0)	11	(5.0–21.0)	29	(14.5–43.0)
	>40	173	8	(2.0–12.0)	9	(4.0–12.0)	5	(2.0–11.0	11	(5.0–20.0)	36	(21.0–48.0)
Sex	Female	116	9.5 *	(2.0–13.0)	9.0	(4.7–13.0)	8.0 *	(3.0–12.0)	12.0 **	(6.0–21.0)	34.0	(21.5–48.5)
	Male	84	4.5 *	(2.0–12.0)	7.5	(3.0–12.0)	5.0 *	(1.7–10.0)	9.0 **	(4.0–12.0)	36.0	(19.5–47.0)
BMI	≤24.9	43	7.0	(1.0–12.0)	8.0	(3.0–12.0)	5.0	(0.5–9.5)	11.0	(4.0–21.5)	23.5 **	(6.5–39.8)
	>25.0	157	9.0	(2.0–12.0)	9.0	(4.0–13.0)	7.0	(3.0–11.0)	11.0	(6.0–20.0)	37.0 **	(23.0–49.2)
Hypertension	Yes	97	7.0	(2.0–12.0)	8.0	(3.0–12.0)	5.0	(2.0–11.0)	9.0	(5.0–18.0)	34.5	(21.5–47.0)
	No	113	9.0	(2.0–12.0)	9.0	(4.0–12.5)	7.0	(3.0–11.0)	12.0	(6.0–20.5)	36.5	(19.0–49.0)
Ischemic heart disease	Yes	24	10.5	(2.7–13.2)	10.5 *	(8.0–14.0)	8.0	(3.7–11.3)	13.0	(9.0–18.3)	40.5	(22.0–46.3)
	No	176	8.0	(2.0–12.0)	8.0 *	(4.0–12.0)	6.0	(2.0–11.0)	11.0	(5.0–20.0)	34.5	(20.0–48.0)
Diabetes	Yes	19	11.0	(7.5–15.0)	14.0 ***	(10.5–16.0)	10.0 *	(4.5–13.5)	21.0 **	(15.0–25.5)	58.0 **	(38.0–65.0)
	No	181	8.0	(2.0–12.0)	8.0 ***	(4.0–12.0)	6.0 *	(2.0–11.0)	10.0 **	(5.0–19.0)	33.0 **	(20.0–47.0)
Any chronic thyroid disease	Yes	35	4.0	(2.0–11.5)	7.0	(3.5–12.0)	4.0	(1.5–10.0)	9.0	(5.0–24.5)	24.0 *	(14.5–36.5)
	No	135	9.0	(2.0–12.0)	9.0	(4.0–13.0)	7.0	(2.0–11.0)	11.0	(5.0–19.0)	37.0 *	(21.5–49.0)
Tobacco smoking	Yes	15	12.0 **	(10.0–13.5)	12.0 **	(11.5–12.5)	11.0 *	(8.5–12.0)	21.0 **	(13.5–22.0)	55.0 **	(49.0–65.0)
	No	185	7.0 **	(2.0–12.0)	8.0 **	(4.0–12.0)	5.0 *	(2.0–11.0)	10.0 **	(5.0–19.0)	33.0 **	(20.0–46.0)

* *p* < 0.05; ** *p* < 0.01; *** *p* < 0.001; HADS—Hospital Anxiety and Depression Scale; GAD-7—Generalized Anxiety Disorder-7; BDI—Beck Depression Inventory; MFIS—Modified Fatigue Impact Scale.

**Table 5:** *p*-Values from Fisher’s exact test evaluating associations between psychological test results and selected clinical risk factors.

Test	Diabetes	Psychiatric Disorders	Tobacco Smoking	Sex
GAD-7	**0.0484**	1.0000	**0.0038**	**0.0115**
HADS—anxiety	0.1466	0.7818	0.0553	**0.0290**
HADS—depression	**0.0023**	**0.0201**	**0.0015**	0.5598
BDI	**0.0029**	0.1704	**0.0064**	**0.0444**

GAD-7—Generalized Anxiety Disorder-7; HADS—Hospital Anxiety and Depression Scale; BDI—Beck Depression Inventory.

**Table 6:** The probability of developing anxiety and depression in post-COVID-19 syndrome participants.

Psychological Test	Comorbidities				Probability
	Diabetes	Tobacco Smoking	Sex	Pre-Existing Psychiatric Disorders	
HADS—anxiety		0	0		0.306
		0	1		0.461
		1	0		0.566
		1	1		0.717
HADS—depression	0	1		0	0.764
	0	0		1	0.698
	0	1		1	0.946
	1	0		0	0.683
	1	1		0	0.942
	1	0		1	0.921
	1	1		1	0.989
GAD-7	0	0	0		0.205
	0	0	1		0.393
	0	1	0		0.564
	0	1	1		0.764
	1	0	0		0.454
	1	0	1		0.676
	1	1	0		0.806
	1	1	1		0.912
BDI	0	0	0		0.287
	0	0	1		0.469
	0	1	0		0.663
	0	1	1		0.812
	1	0	0		0.718
	1	0	1		0.848
	1	1	0		0.926
	1	1	1		0.965

HADS—Hospital Anxiety and Depression Scale; GAD-7—Generalized Anxiety Disorder-7; BDI—Beck Depression Inventory. Diabetes: Yes = 1, No = 0; Tobacco smoking: Yes = 1, No = 0; Sex: woman = 1, man = 0; Psychiatric disorders Yes = 1; No = 0.

## Data Availability

The data presented in this study are available from the corresponding author upon request. The data are not publicly available due to ethical restrictions, in particular those related to the protection of personal data and the privacy of the study participants.

## Supplementary Materials

The following supporting information can be downloaded at: https://www.mdpi.com/article/10.3390/medicina62071234/s1, Table S1: *p*-Values from Fisher’s exact test evaluating associations between psychological test results and all clinical risk factors; Table S2: *p*-Values from Fisher’s exact test evaluating associations between all variables; Table S3: Logistic regression analysis for GAD-7 (dependent variable); Table S3a: Odds ratios and 95% confidence intervals from logistic regression analysis for GAD-7 Variable; Table S4: Logistic regression analysis for HADS-A (dependent variable); Table S4a: Odds ratios and 95% confidence intervals from logistic regression analysis for HADS-A Variable; Table S5: Logistic regression analysis for HADS-D (dependent variable); Table S5a: Odds ratios and 95% confidence intervals from logistic regression analysis for HADS-D Variable; Table S6: Logistic regression analysis for BDI (dependent variable); Table S6a: Odds ratios and 95% confidence intervals from logistic regression analysis for BDI Variable; Table S7: Comparison of the evaluated logistic regression models with the null (intercept-only) model.
