# Supplementary material for: Anxiety-Depressive Disorders in 200 Patients with Post-COVID-19 Syndrome: Prevalence and Predictors from a Cross-Sectional Study
Source: Medicina (Kaunas). 2026 Jun 26;62(7):1234. doi: 10.3390/medicina62071234 (PMC13414012; doi:10.3390/medicina62071234)
Supplement: Supplementary file 1 [file medicina-62-01234-s001.zip › medicina-4335411-supplementary.pdf]

**Table S1.** P-values from Fisher's exact test evaluating associations between psychological test results and all clinical risk factors.

| Test | Hypertension | Ichemic heart disease | Diabetes | Any chronic thyroid disease | Psychiatric disoders | Tobacco smoking | BMI | Sex |
|---|---|---|---|---|---|---|---|---|
| GAD-7 | 0.7691 | 0.3676 | 0.0484 | 0.1775 | 1 | 0.0038 | 0.1091 | 0.0115 |
| HADS-anxiety | 0.5669 | 0.3851 | 0.1466 | 0.0934 | 0.7818 | 0.0553 | 0.3837 | 0.029 |
| HADS-depression | 0.7727 | 0.2741 | 0.0023 | 0.343 | 0.0201 | 0.0015 | 0.8605 | 0.5598 |
| BDI | 0.1575 | 0.6673 | 0.0029 | 0.8521 | 0.1704 | 0.0064 | 0.7299 | 0.0444 |

GAD-7 - Generalized Anxiety Disorder-7; HADS - Hospital Anxiety and Depression Scale; BDI - Beck Depression Inventory. Significance level=0.05.

**Table S2.** P-values from Fisher's exact test evaluating associations between all variables.

| Variables | Hypertension | Ichemic heart disease | Diabetes | Any chronic thyroid disease | Psychiatric disorders | Tobacco smoking | BMI | Sex |
|---|---|---|---|---|---|---|---|---|
| Hypertension | 0 | 0.0281 | 0.4722 | 0.1417 | 0.41 | 1 | 0.0031 | 0.045 |
| Ichemic heart disease | 0.0281 | 0 | 4e-4 | 0.7742 | 1 | 1 | 0.0316 | 0.2701 |
| Diabetes | 0.4722 | 4e-4 | 0 | 0.537 | 1 | 0.1584 | 0.0817 | 0.0551 |
| Any chronic thyroid disease | 0.1417 | 0.7742 | 0.537 | 0 | 1 | 1 | 0.0674 | 3e-4 |
| Psychiatric disorders | 0.41 | 1 | 1 | 1 | 0 | 0.6057 | 0.5055 | 0.7811 |
| Tobacco smoking | 1 | 1 | 0.1584 | 1 | 0.6057 | 0 | 0.5324 | 0.5915 |
| BMI | 0.0031 | 0.0316 | 0.0817 | 0.0674 | 0.5055 | 0.5324 | 0 | 0.0052 |
| Sex | 0.045 | 0.2701 | 0.0551 | 3e-4 | 0.7811 | 0.5915 | 0.0052 | 0 |

Significance level=0.05.

**Table S3.** Logistic regression analysis for GAD-7 (dependent variable).

| | Estimate | Std. Error | z value | p-value |
|---|---|---|---|---|
| Intercept | -1.3546 | 0.2822 | -4.801 | <0.001 |
| Sex | 0.9195 | 0.3322 | 2.768 | 0.0056 |
| Tobacco smoking | 1.6111 | 0.6252 | 2.577 | 0.0100 |
| Diabetes | 1.1689 | 0.5327 | 2.194 | 0.0282 |

Model fit statistics: AIC = 250.2; Null deviance = 262.5 (df = 199); Residual deviance = 242.2 (df = 196).

**Table S3. a.** Results of logistic regression analysis for GAD-7 Variable OR 95% CI p-value.

| Variable | OR | 95% CI |
|---|---|---|
| Sex | 2.51 | 1.33–4.89 |
| Tobacco smoking | 5.01 | 1.57–19.3 |

| | | |
|---|---|---|
| Diabetes | 3.22 | 1.14–9.46 |

**Table S4.** Logistic regression analysis for HADS-A (dependent variable).

| Variable | Estimate | Std. Error | z value | p-value |
|---|---|---|---|---|
| Intercept | -0.8192 | 0.2389 | -3.429 | 0.0006 |
| Sex | 0.6616 | 0.3013 | 2.196 | 0.0281 |
| Tobacco smoking | 1.0863 | 0.5746 | 1.891 | 0.0587 |

Model fit statistics: AIC = 268.35; Null deviance = 271.45(df = 199); Residual deviance = 262.35 (df = 197).

**Table S4. a.** Results of logistic regression analysis for HADS-A Variable OR 95% CI p-value.

| Variable | OR | 95% CI |
|---|---|---|
| Sex | 1.94 | 1.08–3.53 |
| Tobacco smoking | 2.96 | 0.99–9.95 |

**Table S5.** Logistic regression analysis for HADS-D (dependent variable).

| Variable | Estimate | Std. Error | z value | p-value |
|---|---|---|---|---|
| Tobacco smoking | 2.0258 | 0.6769 | 2.993 | 0.0028 |
| Diabetes | 1.6185 | 0.5612 | 2.884 | 0.0039 |
| Psychiatric disorders | 1.6884 | 0.6203 | 2.722 | |

Model fit statistics: AIC = 248.12; Null deviance = 268.37 (df = 199); Residual deviance = 240.12 (df = 196).

**Table S5. a.** Results of logistic regression analysis for HADS-D Variable OR 95% CI p-value.

| Variable | OR | 95% CI |
|---|---|---|
| Tobacco smoking | 7.58 | 2.23–34.8 |
| Diabetes | 5.05 | 1.76–16.6 |
| Psychiatric disorders | 5.41 | 1.70–20.6 |

**Table S6.** Logistic regression analysis for BDI (dependent variable).

| Variable | Estimate | Std. Error | z value | p-value |
|---|---|---|---|---|
| Tobacco smoking | 1.5862 | 0.6839 | 2.319 | 0.02038 |
| Diabetes | 1.8468 | 0.6110 | 3.023 | 0.0025 |
| Sex | 0.7843 | 0.3159 | 2.483 | 0.01302 |

Model fit statistics: AIC = 260.48; Null deviance = 275.64 (df = 199); Residual deviance = 252.48 (df = 196).

**Table S6 a.** Results of logistic regression analysis for BDI Variable OR 95% CI p-value.

| Variable | OR | 95% CI |
|---|---|---|
| Tobacco smoking | 4.89 | 1.42–22.7 |
| Diabetes | 6.34 | 2.07–24.0 |
| Sex | 2.19 | 1.19–4.12 |

**Table S7.** Comparison of the evaluated logistic regression models with the null (intercept-only) model.

| Model | AIC model | AIC null | LRT p-value |
|---|---|---|---|
| GAD-7 | 250.2 | 264.5 | 0.0001 |
| HADS-A | 268.35 | 273.5 | 0.0106 |
| HADS-D | 248.12 | 270.37 | 0.0000 |
| BDI | 260.48 | 277.64 | 0.0000 |
